# Cerebral Revascularization for the Management of Symptomatic Pure Arterial Malformations

**DOI:** 10.3389/fneur.2021.755312

**Published:** 2021-12-16

**Authors:** Xiaocheng Lu, Xinggen Fang, Yabo Huang, Peng Zhou, Zhong Wang, Waleed Brinjikji, Gang Chen

**Affiliations:** ^1^Department of Neurosurgery, The First Affiliated Hospital of Soochow University, Suzhou, China; ^2^Department of Neurosurgery, Wannan Medical College, Yijishan Hospital, Wuhu, China; ^3^Departments of Radiology, Mayo Clinic, Rochester, MN, United States; ^4^Departments of Neurosurgery, Mayo Clinic, Rochester, MN, United States

**Keywords:** pure arterial malformations, cerebral revascularization, systematic review, EC-IC bypass, proximal occlusion

## Abstract

**Background:** Pure arterial malformations (PAMs) are extremely rare abnormalities defined as dilated, overlapping, and tortuous arteries with a coil-like appearance in the absence of venous components. Over the last half century, only seven published reports have described cases of patients with PAMs who received treatment.

**Methods:** Here, we report two cases of women with PAMs who received surgical treatment, and we present a systematic review of the literature. We searched the PubMed, Embase, Web of Science, and Medline databases (up until October 1, 2021) for relevant publications. We performed independent-sample *t*-tests and Fisher's exact tests to compare continuous and categorical characteristics among the available cases.

**Results:** Our first patient was a 43-year-old woman with PAM of the left internal carotid artery (ICA), who received an ICA-radial artery (RA)-M2 bypass. Post-operative digital subtraction angiography (DSA) revealed the disappearance of the left ICA PAM without ischemic events during follow-up. The second patient was a 53-year-old woman with PAMs of the right ICA and posterior cerebral artery. The P1 lesion was treated by proximal occlusion combined with a superficial temporal artery-P2 bypass. During the 12-month follow-up period, the size of the PAMs decreased significantly as indicated by the post-operative DSA showing the absence of hemorrhages. Our systematic review, which includes 56 PAMs, shows that the reported PAMs were more common in the anterior circulation (33/56, 58.9%) than in the posterior circulation (11/56, 19.7%). Bilateral PAMs were more likely to affect bilateral anterior cerebral arteries (ACA) (ACA_bilateral_ vs. ACA_unilateral_: 63.6 vs. 26.2%, *p* = 0.02). In addition, PAMs involving the anterior circulation were likely to affect multiple arteries (anterior_multi_ vs. posterior_multi_: 30.3 vs. 0%, *p* = 0.038).

**Conclusion:** We found very few reports on treated PAMs; further studies with large sample sizes and long follow-up periods are required to explore the appropriate treatment strategy for PAMs.

## Introduction

Pure arterial malformations (PAMs) are extremely rare abnormalities defined as dilated, overlapping, and tortuous arteries with a coil-like appearance in the absence of any venous component. These lesions are first defined by McLaughlin et al. (1) and are often mistaken for arteriovenous malformations (AVMs) or dissecting intracranial aneurysms. PAMs are thought to follow a benign natural progression, and patients with asymptomatic PAMs are managed conservatively. However, we only found reports on seven symptomatic patients who received treatment (2–7). Coil embolization or surgical clipping of aneurysms associated with PAMs were reported on 5 patients, and management of the PAM was performed on 2 patients. Here, we report two new cases of symptomatic PAMs: one who presented with intermittent syncope and one with sudden loss of consciousness. The symptoms were thought to be associated with PAMs, and the patients received PAM surgical treatments. Our analysis suggests that proximal occlusion of PAMs, combined with EC-IC bypass, is a promising method for the treatment of symptomatic PAMs. However, no comprehensive systematic review focusing on PAMs has been published. Therefore, we performed a detailed review of the existing literature to better characterize the clinical characteristics of PAMs.

## Methods

### Literature Search and Inclusion Criteria

We followed the Preferred Reporting Items for Systematic Reviews and Meta-Analyses (PRISMA) guidelines to perform our systematic review (8). We searched the electronic PubMed, Embase, Web of Science, and Medline databases (last search update was on October 1, 2021) using the following search terms: “pure arterial anomalies” OR “pure arterial malformation” OR “dilated” OR “overlapping” OR “tortuous” OR “coil-like” OR “arterial loops” AND “ACA” OR “anterior cerebral artery” OR “MCA” OR “middle cerebral artery” OR “ICA” OR “internal carotid artery” OR “PCA” OR “posterior cerebral artery” OR “PCoA” OR “posterior communicating artery.” In addition, we manually checked the references of all retrieved articles for potential additional studies.

### Study Eligibility and Data Extraction

We included all studies reporting on patients diagnosed with PAMs and meeting the definition put forth by McLaughlin. For each eligible study, two authors independently extracted the following data: first author name, date of publication, study design, sample size, patient characteristics (such as sex and age of patients, associated symptoms, neurological examinations), PAM characteristics (such as maximum diameter and location), imaging appearance, and follow-up duration.

### Statistical Analysis

Continuous variables are expressed as means ± standard deviations (SDs), and categorical variables as numbers (%). We performed independent-sample *t*-tests and Fisher's exact tests to compare continuous and categorical characteristics, respectively. We considered differences as significant when the *P*-value was lower than 0.5. We analyzed all data using SPSS 19.0 (SPSS, Chicago, IL, United States).

## Results

### Case 1

**Figure 1:**
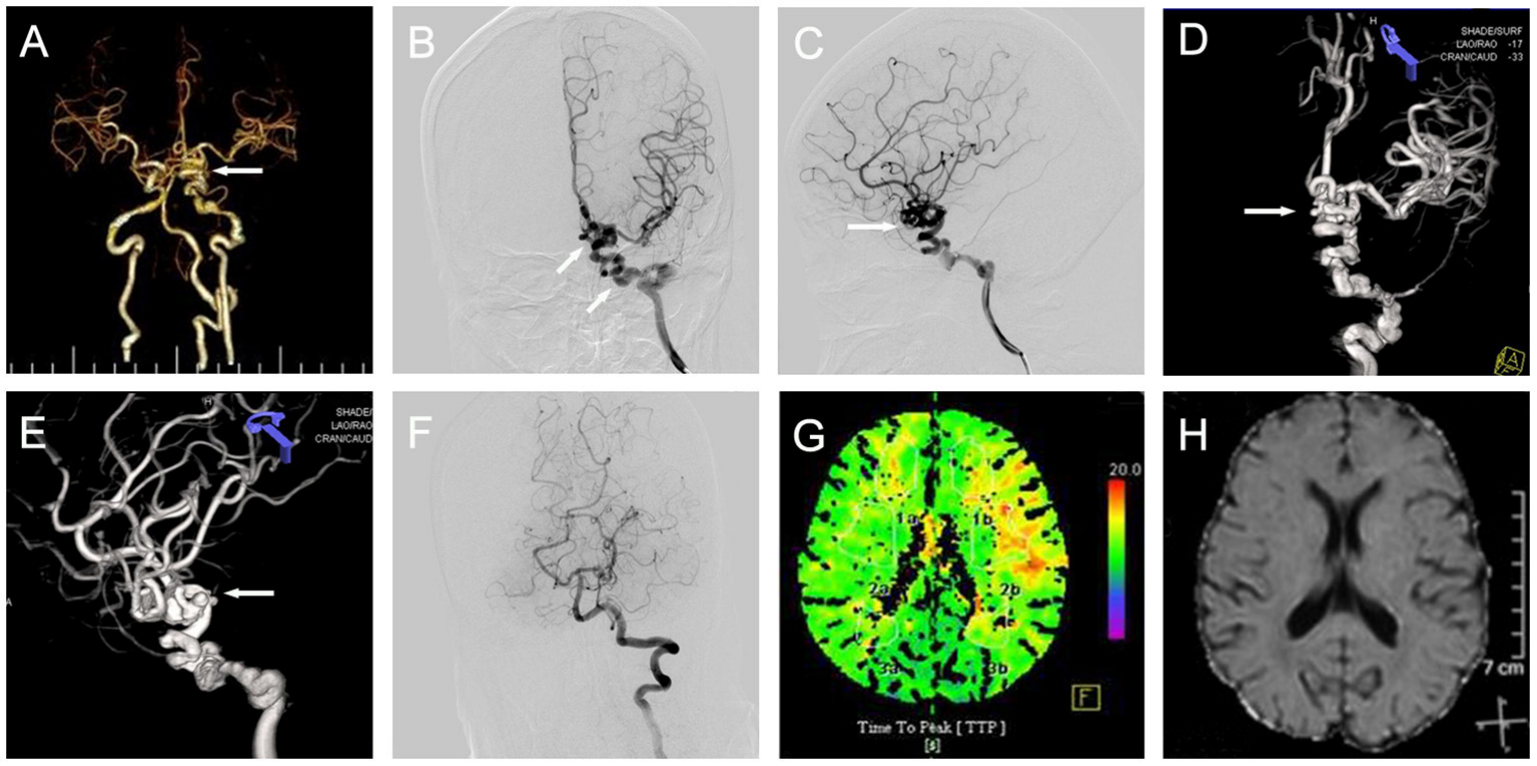
Case 1. Three-dimensional reconstruction of CT angiogram showing a dilated, overlapping, and tortuous [**(A)**, arrow] left ICA, [**(B)**, arrow] anteroposterior, [**(C)**, arrow] and lateral DSA views; and three-dimensional anteroposterior [**(D)**, arrow], and lateral [**(E)**, arrow] DSA views. **(F)** Anteroposterior view of left vertebral artery injection angiograms. **(G)** Axial CTP showing increased TTP in the left hemisphere. **(H)** Axial DWI MRI showing the absence of ischemic stroke. ICA, internal carotid artery; CT, computed tomography; DSA, digital subtraction angiography; DWI, diffusion-weighted imaging; MRI, magnetic resonance imaging; TTP, time to peak; CTP, computed tomography perfusion.

A 43-year-old woman presented with intermittent syncope that had lasted for 10 days, and mild right-sided weakness for 1 week. CT angiogram revealed a dilated and tortuous left internal carotid artery (ICA) with a coil-like appearance (Figure 1A). To better characterize this vascular lesion, we ordered a digital subtraction angiography (DSA), which revealed a dilated, overlapping, and tortuous left ICA (from the petrous segment to the supraclinoid segment), indicating PAM of the left ICA (Figures 1B–F). A computed tomography perfusion (CTP) showed a delayed time to peak (TTP) in the left hemisphere when compared with that in the right hemisphere (Figure 1G). Next, we ordered an MRI to exclude ischemic stroke. The results of the DWI MRI revealed the absence of an acute ischemic stroke (Figure 1H). Together, these results demonstrated that PAM of the left ICA led to decreased blood flow velocity in the left hemisphere, which caused the right-sided weakness and repeated TIAs in our patient.

**Figure 2:**
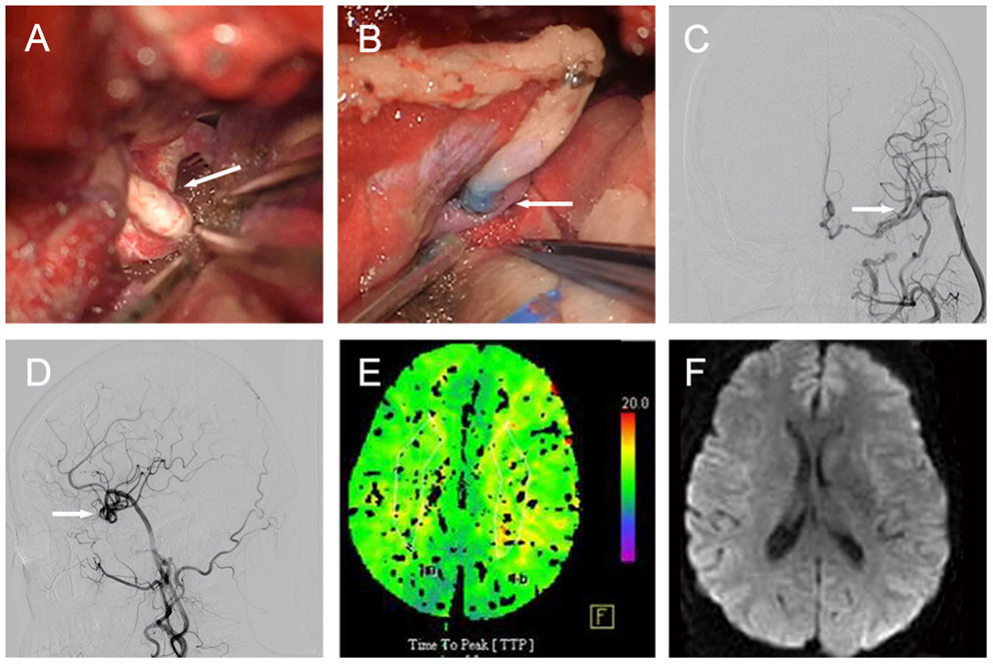
Case 1. [**(A)**, arrow] Intraoperative image showing the left ICA constructed of double loops. [**(B)**, arrow] PAMs treated by ligation of cervical ICA and ICA-RA-M2 bypass. Post-operative DSA demonstrating PAM disappearance and patent bypass in [**(C)**, arrow] anteroposterior, and [**(D)**, arrow] lateral views. **(E)** Axial CTP showing similar TTPs between the two hemispheres. **(F)** Axial DWI MRI evidencing the absence of ischemic stroke. ICA, internal carotid artery; DSA, digital subtraction angiography; PAM, pure arterial malformation; RA, radial artery; DWI, diffusion weighted imaging; MRI, magnetic resonance imaging; TTP, time to peak; CTP, computed tomography perfusion.

Then, the patient agreed to undergo surgical treatment. During the operation, we found that the ectatic vessel of the left ICA comprised double loops (Figure 2A). Briefly, we harvested the radial artery (RA) and prepared it to be anastomosed in an end-to-side fashion to the M2 segment of the middle cerebral artery (MCA). Subsequently, we ligated the distal end of cervical ICA, and anastomosed the proximal end of the cervical ICA to the free end of the RA (Figure 2B). Following the bypass, we confirmed the vessel's patency with indocyanine green video angiography and a Doppler ultrasonic probe. A post-operative DSA (6 months after the operation) revealed that the dilated, overlapping, and tortuous left ICA had disappeared, and that the bypass was patent (Figures 2C,D). A CTP showed similar TTPs between the two hemispheres (Figure 2E), and the DWI MRI confirmed the absence of ischemic stroke (Figure 2F). Moreover, the patient's preoperative symptoms disappeared during the 26-month follow-up.

### Case 2

**Figure 3:**
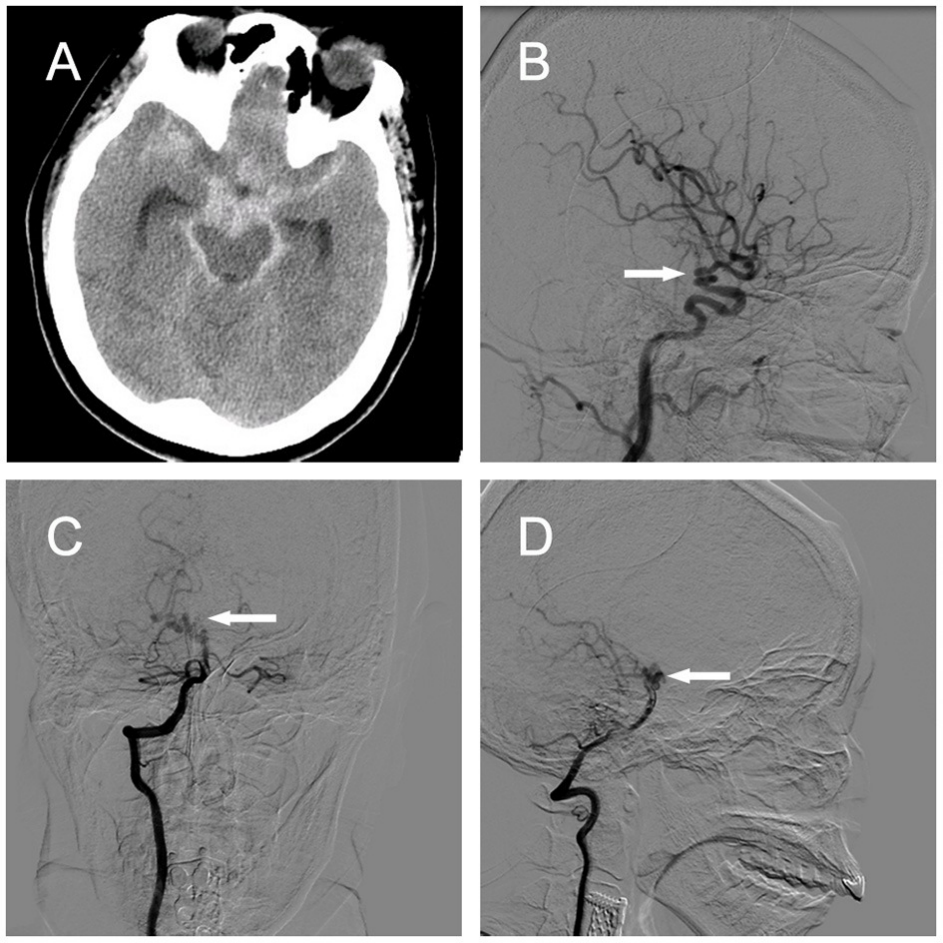
Case 2. **(A)** Head CT revealing SAH in the suprasellar cistern, ambient cistern, and left sylvian fissure. [**(B)**, arrow] Right ICA angiograms showing a coiled and tortuous vessel of the right supraclinoid ICA in the lateral view. Right vertebral artery angiograms demonstrating a coiled and tortuous right P1 segment in **(C)** anteroposterior and [**(D)**, arrow] lateral views. ICA, internal carotid artery; CT, computed tomography.

**Figure 4:**
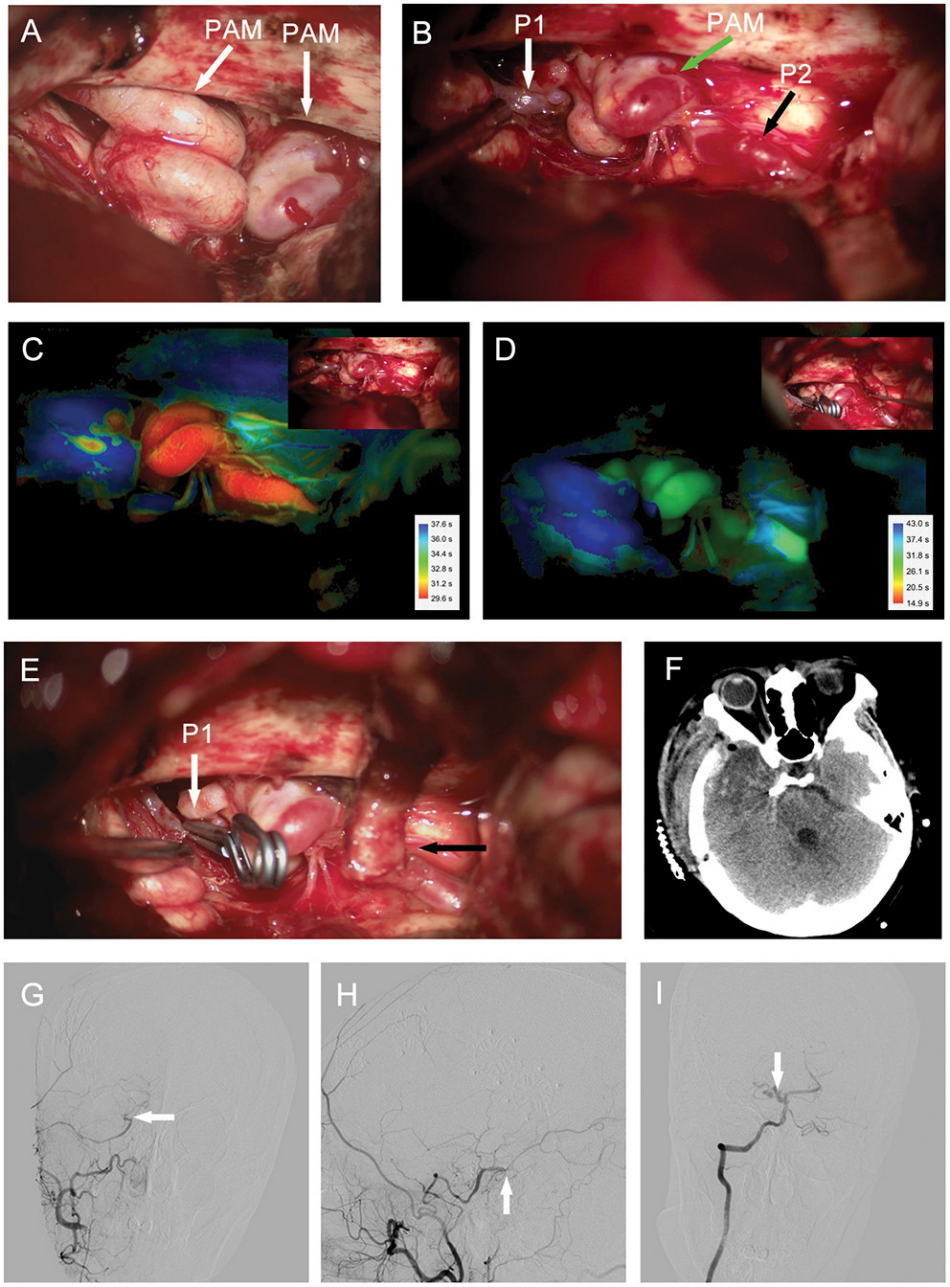
Case 2. **(A,B)** Intraoperative photograph showing **(A)** right ICA and **(B)** right P1 segment lesions composed of 2–3 tightly coiled loops (arrows). Intraoperative ICG video angiography analyzed with Flow800 revealing decreased perfusion in the lesion of P1 segment after proximal occlusion and bypass **(D)**; compared to the image before proximal occlusion **(C)**. Vascular lesion treated by [**(E)**, white arrow] proximal occlusion of the P1 segment and [**(E)**, black arrow] STA-P2 bypass. **(F)** Post-operative head CT operative region. Post-operative DSA showing bypass patency in [**(G)**, arrow] anteroposterior and [**(H)**, arrow] lateral views. [**(I)**, arrow] Right vertebral artery angiograms demonstrating that the P1 segment lesion is smaller than the preoperative lesion in the anteroposterior view. CT, computed tomography; DSA, digital subtraction angiography; ICA, internal carotid artery; STA, superficial temporal artery.

A 53-year-old woman with severe headache that lasted for 1 week was brought to the emergency department because of sudden loss of consciousness. Head computed tomography (CT) revealed subarachnoid hemorrhage (SAH) spanning the suprasellar and ambient cisterns, and the left sylvian fissure (Figure 3A). Cerebral CT angiogram showed vascular lesions in the right ICA and right P1 segment of the posterior cerebral artery (PCA) (data not shown). Subsequent DSA demonstrated dilated, tortuous, and redundant right vessels of the supraclinoid ICA (Figure 3B) and the right P1 segment (Figures 3C,D). Neurological examination revealed decreased consciousness and stiff-neck. Given these findings, we considered the lesions in the right supraclinoid ICA and right P1 segment of the PCA as PAMs, with the latter probably causing the SAH. We decided to treat the patient surgically. During the operation, we found that the right ICA lesion was composed of 2–3 tightly coiled loops with stiff vessel walls, but without bleeding. The lesion in the P1 segment had a similar phenotype and was located on the surface of the midbrain with several branch arteries arising from the lesion to supply the brainstem. In addition, we found fresh blood clots around the P1 lesion and in the ambient cistern (Figures 4A,B).

We treated the P1 segment lesion by proximal occlusion combined with STA-P2 bypass. We confirmed the patency of bypass intraoperatively by Doppler ultrasonic probe and ICG video angiography. Moreover, the intraoperative ICG video angiography analyzed with Flow800 showed a blue P1 segment lesion after the proximal occlusion and STA-P2 bypass, indicating decreased perfusion of the lesion after the surgical procedure compared with perfusion before the treatment (Figures 4C–F). Post-operative CTA demonstrated that the bypass was patent (Figures 4G,H). The P1 segment lesion was still present after the operation but was smaller than the preoperative lesion according to the DSA images (Figure 4I). The patient's neurological examination was almost normal at discharge and remained so during the 12-month follow-up period.

### System Review

**Figure 5:**
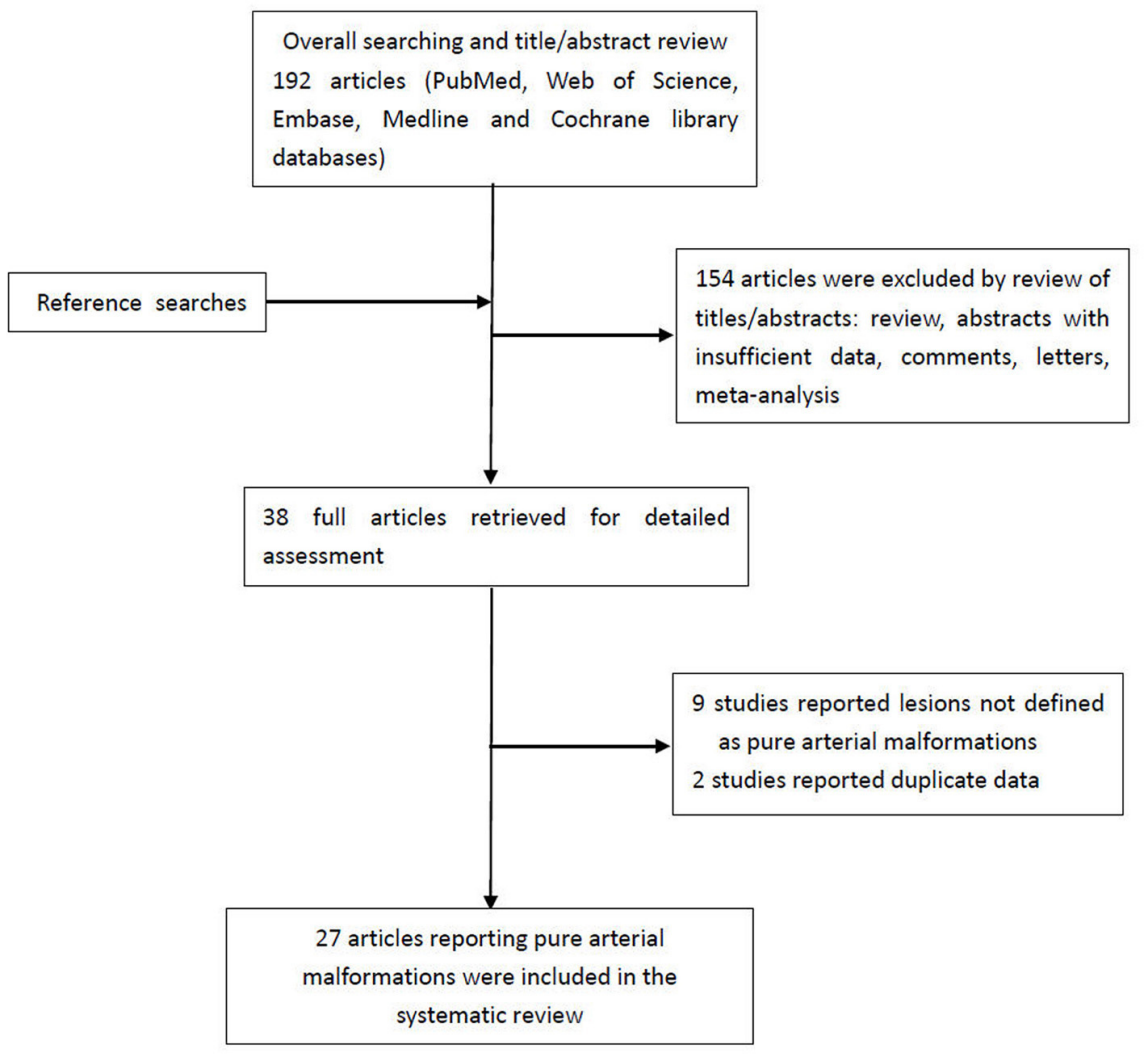
Flow chart of the literature search for systematic review.

A flowchart detailing the process of study selection is shown in Figure 5. Briefly, the literature search produced 192 articles, of which 154 were excluded by review of the abstracts. Thereafter, full texts of the remaining 38 articles were analyzed and reviewed in detail. Finally, our literature search yielded 27 studies with 56 patients (including the two patients in this study) confirmed as having PAMs and meeting the definition put forth by McLaughlin et al. (1, 2, 4–7, 9–29) (Supplementary Table 1). Table 1 summarizes the main characteristics of all studies included in this system review. The average age of patients at the time of presentation with PAMs was 30.9 ± 17.3 years, and PAMs were more common among women (37/56, 66.1%). Studies have shown that PAMs affect all segments of the intracranial arteries, such as the ACA, PCA, PCoA, and supraclinoid ICA. However, our analysis suggests that PAMs are more common in the anterior circulation (33/56, 58.9%) than in the posterior circulation (11/56, 19.7%). Moreover, PAMs involving both the anterior and posterior circulations were seen in 12 patients (12/56, 21.4%). The majority of lesions affected a single vessel (34/56, 60.7%), and up to 5 vessels could be affected.

**Table 1:** Clinical characteristics of 56 patients diagnosed with PAM.

**Characteristics**	**Value**
**Age**	30.9 ± 17.3
**Sex**
Female	37 (66.1%)
Male	19 (33.9%)
**Side**
Left	24 (42.9%)
Right	18 (32.1%)
Bilateral	11 (19.6%)
Midline	3 (5.4%)
**Lesion location**
Anterior circulation	33 (58.9%)
Posterior circulation	11 (19.7%)
Both anterior and posterior circulation	12 (21.4)
**Number of involved vessels**
1 vessel	34 (60.7%)
2 vessels	9 (16.1%)
3 vessels	9 (16.1%)
4 vessels	1 (1.8%)
5 vessels	3 (5.3%)
**Follow-up (months)**

*PAM, pure arterial malformation*.

Interestingly, in the anterior circulation, PAMs were more likely to affect the ICA, MCA, and ACA; while PCA seems more likely to be affected in the posterior circulation. Moreover, we also found 11 patients with bilateral PAMs (19.6%), which were more likely to affect bilateral ACA as opposed compared to unilateral PAMs (ACA_bilateral_ vs. ACA_unilateral_: 63.6 vs. 26.2%, *p* = 0.02; Supplementary Table 2). In addition, compared with the PAMs involving the posterior circulation, the PAMs involving the anterior circulation were likely to affect multiple arteries (anterior_multi_ vs. posterior_multi_: 30.3 vs. 0%, *p* = 0.038; Supplementary Table 3).

Among all the reported cases, there were only seven cases received endovascular or surgical treatments (Table 2), of which: the first case reported by Hanakita et al. received an EC-IC bypass of the affected vessel (2). A 42-year-old man underwent a procedure to surgically trap the parent artery of the PAM in the right ACA (5). In addition, the other five patients described by Lanzino et al., Munich et al., Li et al., and Yao et al., underwent coil embolizations, surgical clipping or trapping of aneurysms associated with the PAMs (3, 4, 6, 7).

**Table 2:** Summary of patients with PAM who received treatment.

**References**	**Age, sex**	**PAM location**	**Associated symptoms**	**Treatment**	**Follow-up**
Hanakita et al. (2)	43, female	Rt distal ICA, proximal M1, and Lt PCA	Dysarthria	EC-IC bypass and wrapped ectatic vessel with muscle	None
Lanzino et al. (3)	10, female	Supraclinoid ICA	Headache	Coil embolization of the larger, saccular-type, pseudoaneurysm component at the posterior communicating/posterior cerebral artery	3 years
Munich et al. (4)	37, female	ACoAand BA (superior to basilar apex)	Headache, blurry vision and a partial left CN III palsy	Surgical clipping of the associated aneurysms at the PCA	1 month
Yue et al. (5)	42, male	Rt ACA	Headache	Surgical trapping	3 months
Li et al. (6)	77, male	Lt AICA	SAH	Surgical trapping the associated aneurysm	1.5 months
Yao et al. (7)	51, male	Rt PICA	SAH	Surgical clipping of the associated aneurysm	9 months
	48, male	Rt the first branch of intracranial segment of vertebral artery	SAH	Surgical clipping of the associated aneurysm	3 months
Present study	43, female	Lt ICA	Right-sided weakness	EC-IC bypass (high flow) and ligation of ICA (cervical segment)	26 months
	45, female	Rt ICA and P1	SAH	Proximal occlusion of P1 segment and STA-P2 bypass	12 months

*ACoA, anterior communicating artery; AICA, anterior inferior cerebellar artery; BA, basilar artery; CN, cranial nerve; EC-IC, extracranial-intracranial; ICA, internal carotid artery; Lt, left; PAM, pure arterial malformation; PCA, posterior cerebral artery; Rt, right; SAH, subarachnoid hemorrhage; STA, superficial temporal artery*.

## Discussion

Pure arterial malformations (PAMs), first defined by McLaughlin et al. are extremely rare, and they get commonly mistaken for dissecting intracranial aneurysms and brain AVMs (1, 25, 28). Distinguishing PAMs from other vascular abnormalities (such as AVMs, intracranial arterial dissections, and intracranial dolichoectasia) is important, because treatments vary according to the different types of vascular abnormality. AVM is defined as an abnormal connection between arteries and veins that lacks capillaries. When anomalous interconnections are between dural (pial) arteries and dural (pial) veins, they are known as dural or pial arteriovenous fistulas. Catheter angiograms reveal a nidus or a venous component in arteriovenous malformations or fistulas that is absent in PAMs (30, 31). Moreover, PAMs are distinct from intracranial dolichoectasias, which are cerebral arteries with increased diameter, elongation, and tortuosity. Dolichoectasias involve predominantly the vertebrobasilar artery and ICA, and they usually present as dilated and elongated vessels that remain recognizable in catheter angiograms (32–35). In contrast, PAMs involve impacted overlapped vessels with severe tortuosity that results in a coil-like appearance.

The pathogenesis of PAMs remains unclear with two main potential etiologies: (1) congenital defect that results in arterial dysplasia and (2) viral infection affecting a vulnerable arterial segment. In some report, the arterial dysplasia in PAMs is congenital. Araki et al. reported on a 23-year-old woman diagnosed as having right hemimegalencephaly with DSA and dynamic CT images showing dilatations of the right ACA, MCA, and PCA, as well as increased circulation volume in the right hemisphere (probably accounting for the ipsilateral hemimegalencephaly) (14). Moreover, we found at least five cases of PAMs accompanying white matter or cortical dysplasias in the territory of the affected vessels (10, 16, 19, 28). Neurovascular development, such as the initial formation of neurovascular and ingression of vessel sprouts into the neural tissue, is stimulated by the development of the brain parenchyma (36). Thus, PAMs may be associated with cortical dysplasias. Viral infection may be another potential etiology of PAMs. Lasjaunias showed serial images indicating the development of a tightly coiled PAM in the supraclinoid ICA and M1 segments of a patient with varicella vasculopathy (37). Moreover, a previous study has reported on a 2-year-old boy with a PAM in the A2 segment who died of viral encephalitis (9). Thus, some supraclinoid ICA and MCA PAMs may result from viral infections.

Approximately 85% of the PAMs reported were incidental findings in previous studies. In a recent case series (involving 25 patients diagnosed with PAMs), Oushy et al. reported that these lesions were generally asymptomatic and likely have a benign natural history (28), and that only three of the patients had symptoms potentially associated with their PAMs and 2 patients received treatment (coil embolization) of aneurysms associated with PAMs. In the systematic review, the results showed that the mean age of 9 patients who received treatments was 44 ± 17.2 years. However, patients with untreated PAMs in previously reported studies have been younger (28.4 ± 16.4 years). These patients had a mean follow-up of 32.5 months (including the case of a patient with a 30-year follow-up) or 24.9 months (excluding that patient). Considering the scarcity of cases and the short-term follow-ups in the literature, we speculate that longer-term follow-ups will disclose increased risks of associated ischemic or hemorrhagic strokes in patients with PAMs.

To date, 56 cases of PAMs meeting the criteria put forward by McLaughlin et al. have been reported in the literature (1). However, only seven of those patients received treatments. Moreover, coil embolization, and surgical trapping or clipping of the aneurysms associated with the PAMs were performed in 5 patients. Dealing with the PAM itself was only reported on 2 patients. Hanakita et al. (2) wrapped ectatic middle cerebral artery with muscle combined with EC-IC bypass. In the other patient, the PAM located in the right A1 was trapped without bypass, because the distal branches of the ACA was supplied by the contralateral ICA through the anterior communicating artery. In this study, we report the treatment with cerebral revascularization in two patients with symptomatic PAMs. In the first case, the lesions involved the left ICA (from petrous segment to supraclinoid segment); the cervical ICA was ligated and ICA (proximal end of the ligated ICA)-RA-M2 bypass was performed. The post-operative DSA images showed that the dilated and tortuous vessel had disappeared 6 months after the operation, and the patient remained symptom-free during the 26-month follow-up period. To our knowledge, the patient with SAH in our report is the first surgically confirmed case with PAM involving the PCA. During the operation, we occluded the P1 segment proximal to the lesion and performed STA-P2 bypass. After the surgical treatment, the wall tension in the lesion decreased significantly. Moreover, an intraoperative ICG video angiography analyzed with Flow800 showed that the P1 segment lesion was blue after the proximal occlusion and STA-P2 bypass, indicating decreased perfusion of the lesion after the surgical treatment compared with the perfusion before the treatment (38, 39). The post-operative DSA images demonstrated a lesion smaller than the preoperative one, indicating a decreased re-bleeding risk in the future. The patient demonstrated lack of obvious deficits on neurological examinations during the 12-month follow-up period. We suggest that the P1 segment PAM in this patient was additionally supplied by some small arteries not observed in the preoperative angiography.

## Conclusions

Pure arterial malformations (PAMs) are extremely rare lesions that have been considered to have a benign natural progression. Here, we report on EC-IC bypass treatments of two patients with rare symptoms (one with ischemic symptoms and the other with SAH). After the operation, the symptoms were relieved, and the PAMs disappeared completely or were significantly reduced in size. Very few treated cases of PAM have been reported; further studies with large sample sizes and long follow-up periods are required to identify the most appropriate treatment strategy for PAMs.

## Data Availability Statement

The original contributions presented in the study are included in the article/Supplementary Material, further inquiries can be directed to the corresponding author/s.

## Ethics Statement

The studies involving human participants were reviewed and approved by the Ethical Standards of The First Affiliated Hospital of Soochow University (Suzhou, China). The patients/participants provided their written informed consent to participate in this study.

## Author Contributions

YH, PZ, XF, and XL contributed to the surgical treatment and drafted the article. PZ, XL, GC, and ZW participated in case management, data extraction, and data analysis. WB helped us revised the article. All authors contributed to the article and approved the submitted version.

## Conflict of Interest

The authors declare that the research was conducted in the absence of any commercial or financial relationships that could be construed as a potential conflict of interest.

## Publisher's Note

All claims expressed in this article are solely those of the authors and do not necessarily represent those of their affiliated organizations, or those of the publisher, the editors and the reviewers. Any product that may be evaluated in this article, or claim that may be made by its manufacturer, is not guaranteed or endorsed by the publisher.
