# Supplementary material for: Cerebral Revascularization for the Management of Symptomatic Pure Arterial Malformations
Source: Front Neurol. 2021 Dec 16;12:755312. doi: 10.3389/fneur.2021.755312 (PMC8716448; doi:10.3389/fneur.2021.755312)
Supplement: Supplementary file 1 [file Data_Sheet_1.pdf]

Supplementary Table 1. Summary of previously reported patients with PAM

Authors, year	Age, sex	Lesion location	Associated Symptoms
Sacks, 1969	2, male	Bilateral A2	Viral encephalitis
Wolpert, 1972	21, male	Bilateral pericallosal	Seizure, agenesis of corpus callosum
Thompson, 1976	39, male	Distal Lt ACA	Seizure
Tsukamoto, 1985	37, female	Bilateral pericallosal	Mania
Yamada, 1985	17, female	Lt ICA, M1 and ACA	Nausea and vomiting
	40, female	Rt ICA, M1 and ACA	Right sided hemiparesis
Hanakita, 1986	43, female	Rt distal ICA & proximal M1; Lt PCA	Dysarthria
Araki, 1987	25, female	Rt MCA, ACA, & PCA	Rt hemimegalencephaly
Kryst-Widźgowska, 1980	72, female	Bilateral distal ACAs	Aphasia, rt-sided hemiplegia
Doran, 1995	14, female	Bilateral pre-& supracallosal segments of ACAs	Seizure
Abe, 1997	32, male	Lt MCA	Cortical dysplasia, Szs
Kanemoto, 1998	41, female	Lt MCA	Szs, ipsilateral cavernoma
Vanslambrouk, 2000	5, male	Lt ICA, PCoA, PCA, MCA, & Lt SCA	Minimal Rt hemiparesis
Metry, 2001	1, female	Lt MCA & supraclinoid ICA	PHACE syndrome
Beringer, 2004	49, male	Bilat pericallosal	Intermittent frontal HA
Baccin, 2007	4, female	Lt supraclinoid ICA & PCoA	PHACE syndrome, rt-sided hemiparesis
	1, female	Lt supraclinoid ICA, PCoA, P1, Rt supraclinoid ICA	PHACE syndrome, fever, & hypotonia
Uchino, 2003	35, female	Rt SCA	Severe HA
Shankar, 2009	26, female	Distal Rt PCA	Incidental

McLaughlin, 2013	24, female	Lt PCoA & P2	Frequent HAs & dizziness
McLaughlin, 2014	8, female	Lt supraclinoid ICA & proximal M1	Sinus infection
Lanterna, 2014	1, male	Lt PCoA & PCA	Infarct from moyamoya
Feliciano, 2014	42, male	Rt MCA	HA w/ basal ganglia hemorrhage
Sako, 2016	35, male	Lt PICA	Vertigo
Oushy, 2021	47, female	Rt ACA	Incidental
	41, female	Lt Supraclinoid ICA, MCA, BA, PCoA, PCA	Diplopia, ptosis
	17, male	Rt SCA	HA, dizziness
	10, female	Lt Supraclinoid ICA, PCoA, PCA	Severe HA
	11, male	Lt Supraclinoid ICA, PCoA	Incidental
	25, female	Lt ACA	Incidental
	33, female	Lt ACA	Incidental
	19, female	Lt MCA	Incidental
	27, female	BA	HA, Lt numbness
	38, female	Lt PICA	Incidental
	48, male	Rt PCoA	Coma
	51, female	Lt MCA	Incidental
	25, female	Bilateral Supraclinoid ICA, MCA, PCoA	HA
	27, male	Rt ACA	Incidental
	17, female	Rt PICA	Incidental
	21, female	Rt PCoA	HA

	50, female	Rt MCA	Incidental
	46, female	Lt supraclinoid ICA, A1	Incidental
	37, female	Lt MCA	HA
	54, female	Bilateral Rt PCA, supraclinoid ICA, PCoA, BA	Incidental
	12, male	Rt PCA, PCoA; Lt ICA, ACA, MCA	Incidental
	36, female	Rt ACA	HA
	38, female	BA	Incidental
	17, female	Lt supraclinoid ICA, MCA, ACA	seizures
	26, male	Rt supraclinoid ICA, PCoA, MCA	HA
Munich, 2019	37, female	ACoA and BA (superior to basilar apex)	Headache, blurry vision and a partial left CN III palsy
He, 2019	42, male	Rt ACA	Headache
Li, 2020	77, male	Lt AICA	SAH
Yao, 2021	51, male	Rt PICA	SAH
	48, male	Rt the first branch of intracranial segment of vertebral artery	SAH
Present study	43, female	Lt ICA	Right-sided weakness
	45, female	Rt ICA and P1	SAH

ACA: anterior cerebral artery; ACoA: anterior communicating artery; BA: basilar artery; Lt: left; CN: cranial nerve; ICA: internal carotid artery; MCA: middle cerebral artery; Rt: left; PAM: Pure arterial malformations; PCA: posterior cerebral artery; PCoA: posterior communicating artery; PICA: posterior inferior cerebellar artery; SCA: superior cerebellar artery.

Supplementary Table 2. The correlation between side of PAM and artery involved.

Artery involved	Side		P-value
	Bilateral	Unilateral	
ACA	7	11	0.02
Non-ACA	4	31	

ACA: anterior cerebral artery; PAM: pure arterial malformation.

Supplementary Table 3. The correlation between location of PAM and number of arteries involved.

Number of arteries involved	Lesion location		P-vaule
	Anterior circulation	Posterior circulation	
⩾2	10	0	0.038
1	23	11	

PAM: pure arterial malformation.
